# Differentiating among pragmatic uses of words through timed sensicality judgments

**DOI:** 10.3389/fpsyg.2013.00938

**Published:** 2013-12-19

**Authors:** Valentina Bambini, Marta Ghio, Andrea Moro, Petra B. Schumacher

**Affiliations:** ^1^Center for Neurocognition and Theoretical Syntax, Institute for Advanced Study (IUSS)Pavia, Italy; ^2^Laboratorio di Linguistica “G. Nencioni,” Scuola Normale SuperiorePisa, Italy; ^3^Independent Emmy Noether-Research Group, Department of English and LinguisticsJohannes Gutenberg-University Mainz, Germany

**Keywords:** metaphor, figurative language, loose use, pragmatics, sensicality judgments, experimental pragmatics

## Abstract

Pragmatic and cognitive accounts of figurative language posit a difference between metaphor and metonymy in terms of underlying conceptual operations. Recently, other pragmatic uses of words have been accounted for in the Relevance Theory framework, such as approximation, described in terms of conceptual adjustment that varies in degree and direction with respect to the case of metaphor. Despite the theoretical distinctions, there is very poor experimental evidence addressing the metaphor/metonymy distinction, and none concerning approximation. Here we used meticulously built materials to investigate the interpretation mechanisms of these three phenomena through timed sensicality judgments. Results revealed that interpreting metaphors and approximations differs from literal interpretation both in accuracy and reaction times, with higher difficulty and costs for metaphors than for approximations. This suggests similar albeit gradual interpretative costs, in line with the latest account of Relevance Theory. Metonymy, on the contrary, almost equates literal comprehension and calls for a theoretical distinction from metaphor. Overall, this work represents a first attempt to provide an empirical basis for a theory-sound and psychologically-grounded taxonomy of figurative and loose uses of language.

## Introduction

Word meaning is often modified in use, giving rise to a number of loose and figurative uses. These modulations of meaning are thoroughly context-dependent and their description has fallen under the domain of pragmatics. While it is useful to group together non-literal uses, potential differences in the representation and the underlying interpretative mechanisms should be accounted for and considered experimentally. This paper is concerned with the characterization of three pragmatic phenomena, namely metaphor (e.g., “Some theses are marathons”), metonymy (e.g., “No comments from Buckingham Palace”), and approximation (e.g., “Her face is oval”), and whether they exhibit different interpretation costs, which might support and sharpen theoretical distinctions.

### Theoretical accounts on metaphor, metonymy, and approximation

Existing pragmatic and cognitive accounts differ on whether they treat different types of pragmatically enriched meanings as distinct operations or not. In the Gricean framework, figurative expressions such as metaphor, irony, meiosis, and hyperbole are grouped together as cases of flouting the first Maxim of Quality (“Do not say what you believe to be false”), and require the derivation of an implicature (Grice, 1975). The nature of metonymy is not explored, but presumably metonymic expressions would also be described inferentially, either as another case of flouting the first Maxim of Quality or as a tool to adhere to the Maxim of Manner (Egg, 2004).

In more recent times, Relevance Theory has deepened the study of the inferential processes underlying the comprehension of the lexical items, suggesting that grasping the intended meaning of a word requires a process of adjusting the linguistically encoded concept to construct an *ad hoc* concept, i.e., a concept inferentially derived for that occasion of use, whose denotation is broader (i.e., more inclusive) or narrower (i.e., less inclusive) than the denotation of the lexical concept. Consider the utterance “Boris is a man”: in most contexts the lexically encoded concept MAN would result under informative and would require, for instance, to be narrowed down to the *ad hoc* concept MAN^*^ as “ideal man,” in order to reach the intended interpretation. Conversely, in “This policy will bankrupt the farmers,” the encoded concept BANKRUPT could be taken literally, but in certain contexts is likely to require an adjustment that goes in the opposite direction, namely to be broadened in order to include cases in which the farmers are close enough to bankruptcy (Carston, 2009).

In this view, most words require an adjustment process resulting in an *ad hoc* concept, and the difference depends on the direction of the adjustment (broadening or narrowing with respect to the denotation), and also on the degree of it, ranging from less to more context-dependent and occasion-specific uses (Wilson, 2003; Sperber and Wilson, 2005; Wilson and Carston, 2006). Well-studied examples of different degrees of broadening include language uses known in the literature with the labels of approximation, hyperbole, and metaphor. Approximation is a variety of broadening that includes a relatively marginal adjustment of the encoded concept, to cover just a “penumbra” of cases that only strictly speaking fall outside the linguistically-specified denotation (e.g., in “The house is empty,” “empty” may be used to communicate that the house is lacking of furniture). Hyperbole involves a more substantial adjustment of the encoded concepts (e.g., “empty” in the previous example is used to communicate that the house, although furnished, does not have as much furniture as desired). Metaphor is a use of language based on an even more radical broadening of the lexical concept (e.g., “empty” can be used metaphorically to indicate that the house lacks of emotional content).

Recently, Carston and Wearing (2011) proposed a finessing of the relevance-theoretic account, by positing a stronger distinction between metaphor and hyperbole: while concept broadening is required both in metaphor and hyperbole understanding, metaphorical uses would require also additional concept narrowing. Consider the following utterances: “My evening jog with Bill turned into a marathon” and “Writing a thesis was a marathon Jane didn't want to repeat.” When intended hyperbolically, as in the first case, the denotation of the *ad hoc* concept MARATHON^*^ is simply more inclusive (broader) than that of the original lexical concept, involving a relaxing of the length of the episode of running. When intended metaphorically, as in the second example, the word goes through a broadening (in order to include instances of activities that are psychologically demanding and exhausting) combined with narrowing (in order to exclude professional marathons; Carston and Wearing, 2011). Following this idea, it seems reasonable to assume that also approximation differs from metaphor in requiring only (and marginally) concept broadening: if a separation holds between the case of metaphor and the case of a substantial yet not radical broadening such as hyperbole, the separation should hold also between metaphor and a marginal broadening like that required by approximation.

As concerns metonymy, a full description in Relevance Theory terms is still lacking (for a preliminary account see Papafragou, 1996), and is indeed considered as an interesting challenge for pragmatics (Carston, 2010). Following Nunberg's (1995) distinction between reference transfer (e.g., “The ham sandwich wants to pay,” where a nominal expression is used to refer to another nominal expression) and predicate transfer (e.g., “Nixon bombed Hanoi,” where the shift concerns the whole predicate “bombed Hanoi”), Wilson and Carston (2007) suggested that both cases seem to require the construction of an *ad hoc* concept, but only the latter case involves broadening and narrowing, while the former should be accommodated in terms of genuine reference substitution or a real world association (see also Carston, 2010). Here we focus on the reference transfer case, and on the idea that this type of metonymy, while still representing a pragmatic use that requires the construction of an *ad hoc* concept, does not involve the same kind of conceptual adjustment observed for metaphor. The view that at least some cases of metonymy can be described as cases of reference transfer—presumably involving a meaning shift mechanism—is in line with other pragmatic accounts distinguishing between loosening, meaning shift, and free enrichment (Recanati, 2010).

Turning to the framework broadly known as Cognitive Linguistics, both metaphor and metonymy are thought of as conceptual phenomena grounded in general cognition. Yet a difference is assumed to characterize the two. Metaphor is described in terms of mapping between two distinct cognitive domains (e.g., in “Love is a journey,” the source domain JOURNEY is mapped onto the target domain LOVE). Metonymy, on the contrary, is based on mapping within the same cognitive domain (e.g., in “He is reading Shakespeare,” the source domain SHAKESPEARE provides access to its sub-domain SHAKESPEARE'S WRITINGS, which is the target domain; Lakoff and Johnson, 1980; Ruiz de Mendoza Ibáñez, 2007). Furthermore, the mapping is taken to be based on different associative relations: resemblance for metaphor and contiguity for metonymy. Standard types of metonymic mappings are, among others, *part for whole, producer for product, place for institution, object used for user* (Panther and Thornburg, 2007). This list suggests the routinized status of many metonymic mappings, and points in the direction of a close relation between metonymy and grammar. There are indeed grammatical structures that seem to be sensitive to metonymically induced interpretations. For example, in “The author began the book,” the verb's logical structure coerces an interpretation in which a part of an event, *the book*, denotes the whole event, *writing the book*, a phenomenon known as “logical metonymy” (Pustejovsky, 1995; Lascarides and Copestake, 1998).

### Formulating empirical predictions

Do the differences among pragmatic uses brought about in the theoretical literature find experimental support? Evidence from direct comparison of metaphor and metonymy is sparse. In a self-paced reading study, Gibbs (1990) showed that metaphorical referential descriptions are understood more easily than metonymic ones. Developmental psychology, however, points in a different direction: with respect to metaphor, metonymy is acquired earlier and processed more accurately (Rundblad and Annaz, 2010). Similarly, patients exhibit different behaviors in processing metaphors vs. metonymies, associated with different lesion sites, which suggests distinct or at least partially independent neural representation (Bisiacchi et al., 1976; Semenza et al., 1980, 1992; Klepousniotou and Baum, 2005).

In considering how different pragmatic uses are processed, we can nevertheless rely on the extensive literature on metaphor and—to a lesser extent—metonymy. It has been shown that metaphor processing is influenced by many factors, such as context, familiarity, difficulty, novelty (Gibbs, 1994; Giora, 2003; Cardillo et al., 2010). However, when placed in a minimal context and controlled for the other factors, processing metaphorical expressions still requires additional effort measured both at the behavioral level (Cacciari and Glucksberg, 1994; Noveck et al., 2001; Bosco et al., 2009) and in terms of brain response (De Grauwe et al., 2010; Bambini et al., 2011; Bambini and Resta, 2012). Metonymy, on the contrary, has produced mixed results. Neurophysiological and neuroimaging evidence for a difference between metonymy and literal processing has been reported, although with no visible effects in terms of behavioral response (Rapp et al., 2011; Schumacher, 2011). Eye-tracking studies showed no differences compared to literal interpretations when metonymies are licensed by the context and mediated by a common metonymic convention (Frisson and Pickering, 1999; Frisson, 2009). Schumacher (2013) suggests even fine-grained distinctions between metonymic types. As for approximation, to the best of our knowledge it hasn't received empirical consideration up to now. However, it has been shown that other types of loose use, such as hyperbole, are read faster than metaphor (Deamer et al., 2010), lending support to the gradient of meaning extension.

From the perspective of experimental pragmatics (Noveck and Reboul, 2008), we attempted to formulate empirical predictions out of the cognitive pragmatic accounts discussed above. Following Relevance Theory, metaphor and approximation are the result of the same conceptual adjustment process, differing solely in the degree and in the direction of this process. Approximation involves only a marginal broadening, while metaphor involves a wider broadening (Wilson, 2003) or broadening coupled with narrowing (Carston and Wearing, 2011). In this sense, metaphor seems to require more meaning modulation than approximation. It may be hypothesized that more meaning modulation would require higher costs of interpretation, thus, predicting: (i) higher interpretation costs for metaphor and approximation in comparison with literal expressions; (ii) higher interpretation costs for metaphor in comparison with approximation.

Metonymy, on the contrary, seems to rely on specific mechanisms. The hypothesis—to a certain extent shared by Relevance Theory, Cognitive Linguistics, and other frameworks—is that metonymy is supported by conceptual processes different from those involved in metaphor processing, such as meaning shift operations within the same conceptual domain. Re-formulating in processing terms, our prediction is that (iii) metonymy could exhibit different interpretation costs with respect to metaphor and approximation. More specifically, based on previous developmental and neuropsychological evidence, metonymic interpretation could come with no extra cost with respect to literal comprehension, at least when the transfer type is routinized (e.g., *producer for product*).

In order to provide empirical evidence in favor of either a distinction or a unified view of the three phenomena, we compared interpretation availability and costs for metaphor, metonymy, and approximation through a timed sensicality judgment paradigm, where participants are asked to decide quickly if a sentence is meaningful or not, and their performance is measured in terms of accuracy and reaction times. This experimental paradigm seems especially suitable to explore meaning modulation, as it requires subjects not only to access the linguistic items but also to elaborate and interpret their meanings at the level of detail that would distinguish different senses (Klein and Murphy, 2001).

Below we will first present detailed background on the construction of the stimulus material, i.e., a *de novo* built set of Italian metaphors, metonymies and approximations with corresponding literal and anomalous counterparts. Then, we describe a rating study in which all sentences were normed for the major psycholinguistic properties considered in the literature on figurative language, namely meaningfulness, difficulty, cloze probability, and familiarity, in order to obtain a pool of stimuli especially controlled for their interpretability. Finally, we go back to the kinds of conceptual adjustments and how they might result in different sensicality judgment responses.

## Rating study

In building an experimental set of different pragmatic uses, two major issues emerge: first the need to rule out possible confounding effects due to sentential and contextual environment, and second the need to control for a number of psycholinguistic variables that are well known to influence figurative language.

As for the first point, for each of the three pragmatic phenomena under investigation (metaphor, metonymy and approximation) we constructed a set of Italian sentences of the form “That Y verb X,” where X was the word triggering the pragmatic interpretation and was taken as the target word for the experimental measures. Given the well-documented role of context in facilitating figurative language processing (Gibbs, 1994; Schumacher, 2012), context was set to a minimal yet sufficient level for interpretation across sets, in order to allow distinct pragmatic mechanisms to emerge neatly. Literal and anomalous counterparts were created for each set by selecting different subject nouns “Y” or different verbs. All the anomalies contained a world knowledge violation, as classically employed in language research (Kutas and Hylliard, 1980; Cotelli et al., 2007). For the metaphor and approximation sets, the anomalous condition resulted from the clash of two incompatible semantic fields, while in the metonymy set the anomalies are related to selectional properties of the lexicon, and specifically of the verbs (see below for set specific criteria). It is also possible to create world knowledge anomalies where all the elements pertain to the same semantic fields and what is wrong is the combination of them (for example, due to causal effects, i.e., “to dry with water”). However, we left this type of anomaly aside and we concentrated on most common anomalies based on a semantic clash.

Among the many variables involved in figurative language, the three sets were rated for meaningfulness, difficulty, cloze probability, and familiarity. The importance of these variables has been extensively described for metaphor processing (Kintsch and Bowles, 2002; Cardillo et al., 2010), partially addressed for metonymy processing (Frisson and Pickering, 1999), and never explored for approximation. In the perspective of the timed sensicality judgments to be collected afterwards, the main purpose of this rating study was to assess the meaningfulness of the experimental items, i.e., the interpretability of the sense of the utterances, along with their difficulty, i.e., the overall ease of interpretation.

As for familiarity, we aimed at setting a medium level of familiarity for the pragmatic uses, in order to exclude both fully conventionalized expressions—that could be processed as idioms rather than through pragmatic adjustment—and highly creative expressions—that could demand special pragmatic processes or even result in senselessness. The familiarity dimension was differently operationalized for each pragmatic phenomenon under investigation. Following the main literature on metaphor, familiarity was assessed by asking participants to rate frequency of experience for each metaphorical sentence. For the metonymy set, we devised a world-knowledge task to control for both the familiarity of the names used as target words and the familiarity of the metonymic transfer for those names. For the approximation set, a typicality task was used, where participants rated how appropriately the adjectives used as target words X qualify the subject words Y.

Lastly, to ensure that context was kept minimal and equal across conditions, we tested the contextual expectancy of each target word X for each sentence in the triplet and for all sets through a cloze probability task.

### Methods

#### Participants

Eighty-five native speakers of Italian (42 F/43 M, mean age = 26.85 ± 3.80, mean schooling years = 18.02 ± 2.04 years of education) participated in the rating study consisting in questionnaires with different tasks (see Sections *Tasks* and *Procedure* below). Participants were unaware of the aim of the questionnaires and were not informed about the inclusion of figurative language. They gave written consent to participate after receiving an explanation of the procedures, according to the Declaration of Helsinki.

#### Materials

For each phenomenon under consideration (metaphor, metonymy, and approximation) we constructed a set of 48 triplets including sentences with the pragmatic use, literal, and anomalous counterparts (henceforth, for the sake of brevity, the label “pragmatic sentences” will be used to refer to the pragmatic use condition for each set). The triplets were designed according to the criteria below, resulting in a total pool of 432 sentences. Table 1 shows an example of triplets from each set (Metaphor set, Metonymy set, Approximation set).

**Table 1:** **Examples of stimulus triplets for the Metaphor set, the Metonymy set, and the Approximation set**.

	**Pragmatic**	**Literal**	**Anomalous**
Metaphor set	Quelle ballerine sono farfalle	Quegli insetti sono farfalle	Quelle bottiglie sono farfalle
	*Those dancers are butterflies*	*Those insects are butterflies*	*Those bottles are butterflies*
Metonymy set	Quello studente legge Camilleri	Quel giornalista intervista Camilleri	Quel cuoco cucina Camilleri
	*That student reads Camilleri*	*That reporter interviews Camilleri*	*That chef cooks Camilleri*
Approximation set	Quelle gomme sono lisce	Quel marmo è liscio	Quei ristoranti sono lisci
	*Those tires are smooth*	*That marble is smooth*	*Those restaurants are smooth*

*Original Italian; English translation in italics*.

#### Metaphor set

We constructed nominal metaphors where a noun X is the vehicle for the metaphorical meaning (e.g., “Those dancers are butterflies”). For each noun, one literal sentence (e.g., “Those insects are butterflies”) and one anomalous sentence (e.g., “Those bottles are butterflies”) was created. Literal sentences were obtained by using semantically compatible terms, while anomalous sentences resulted from the clash of two semantically non-homogeneous terms. Each sentence was constructed in such a way that the first noun phrase (NP) was a subject and the second was a predicate, that is only canonical copular sentences in the sense of Moro (1997). This was made to exclude inverse copular constructions which would have shifted the focus on the post-copular NP, unbalancing the stimuli: e.g., in the canonical sentence “John was the cause of the riot,” where the subject “John” precedes the predicate “the cause of the riot,” “John” is not necessarily focused, whereas in the inverse sentence “The cause of the riot was John,” where the predicate “the cause of the riot” precedes the subject “John,” “John” is necessarily focused, even if in both cases the linear sequence is one and the same (NP V NP). Plural forms were used in order to avoid predictability effects carried by the gender-marked articles required in the singular forms.

#### Metonymy set

We built a set of producer-for-product metonymies, where proper names of well-known Italian people were metonymically used to refer to objects. Different types of *producer for product* shift were used, such as author for book (e.g., “That student reads Camilleri”), musician for song, designer for manufacture, painter for painting. Proper nouns were used in order to create prototypical metonymic uses, as proper names can be considered clear cases of referring expressions that can be used metonymically. In order to control for the higher difficulty in processing proper as compared to common nouns (Semenza, 2006), we carried out a world knowledge rating tests (see the Section *Tasks* below). In terms of Ruiz de Mendoza Ibáñez taxonomy (2007), all metonymies were of the type target-in-source, i.e., the product is a subdomain of the producer. Each proper name X was also combined with different subject nouns (Ys) and different verbs, resulting once in a literal sentence (e.g., “That journalist interviews Camilleri”) and once in an anomalous sentence (e.g., “That chef cooks Camilleri”). In order to confine metonymic interpretation to the target word, subject nouns and verbs were syntactically and semantically congruent in all conditions. Anomalous sentences resulted from the violation of selectional properties of the lexicon, i.e., animate objects were used for verbs selecting inanimate objects. Names of presently popular Italian people (e.g., Camilleri, Vasco) were chosen instead of very famous people from the past (e.g., Dante, Verdi) in order to reduce conventionality, as it has been suggested that the use of famous names (e.g., Dickens) in the metonymic form might have become lexicalized in ordinary language (Frisson and Pickering, 2007).

#### Approximation set

Among the different cases of approximate uses (Wilson, 2003; Wilson and Carston, 2006), we focused on adjectives. Following the examples provided by Wilson and Carston (2007), four main types of adjectives used in an approximate fashion were included: sense-related (e.g., “Those tires are smooth”), geometric-related (e.g., “Those sunglasses are rectangular”), color-related (e.g., “Those clouds are black”), and negative-related adjectives (e.g., “Those strawberries are tasteless”). For each target word X, we created a literal sentence by selecting a prototypical exemplar having the property described by the adjective (e.g., “Those marbles are smooth”), and an anomalous sentence (e.g., “Those restaurants are smooth”). As in the metaphor set, anomalous condition resulted from the clash of two semantically incompatible terms. And as in the metaphor set, all sentences were copular constructions in order to reduce morphological factors and get both the subject and the predicate implemented with the same category.

#### Tasks

To characterize the sentence stimuli with respect to the major psycholinguistic variables considered in the study of figurative language, we designed a rating study consisting of computer-mediated questionnaires including the following tasks:

***Meaningfulness and difficulty tasks***. We asked participants to rate on a five-point Likert scale how meaningful each sentence was (1 = meaningless; 5 = very meaningful). Each sentence was presented one at a time, and participants selected the value of the scale representing their judgment. Next, participants were asked to rate how difficult it was to rate the meaningfulness for that item, on a scale from 1 (very easy) to 5 (very difficult). All sets were tested.

***Familiarity task***. For each item in the Metaphor set, participants were instructed to indicate the frequency of experience with the sentence on a Likert scale from 1 (very unfamiliar) to 5 (very familiar).

***World knowledge task***. For each proper name used in the Metonymy set, participants were instructed to associate the proper name with the corresponding product, choosing between four options. The options vary according to the type of metonymy (e.g., for Camilleri, the options were: book/song/movie/painting). This should account for both the familiarity of the proper names and the familiarity of the producer-for-product transfer.

***Typicality judgments task***. We asked participants to indicate how appropriate a given adjective (e.g., “smooth”) is to qualify three different nouns (e.g., “marble,” “tires” and “restaurants”), which corresponded to the nouns used in the triplet. A 5-point Likert scale (1 = very inappropriate; 5 = very appropriate) was available for each noun. This should assess both the familiarity of the approximate use and the literal use.

***Cloze probability task***. Each sentence was truncated before the target word, and participants were asked to complete with the first word that came to mind. All sets were tested.

#### Procedure

To preserve a high level of attention and avoid fatigue, two different questionnaires were created. Questionnaire 1 included three tasks: meaningfulness coupled with difficulty, world knowledge, and typicality. Questionnaire 2 included cloze probability and familiarity tasks. For each questionnaire, the pool of 432 sentences was inserted into six different lists. Number of pragmatic, literal, and anomalous sentences from each set was equally subdivided in the different lists and tasks. The lists were rotated among tasks so that each sentence was judged only once by each participant. The order of the tasks was counterbalanced across participants using a Latin Square procedure. Within each task the order of sentences was randomized. One group of the participants completed one of the six lists of Questionnaire 1, the other group completed one of the six lists of Questionnaire 2 (number of data points per item per task ≥ 6).

Ratings were administered online through Survey Monkey software (SurveyMonkey.com, LCC, Palo Alto, California, USA, www.surveymonkey.com). Each participant completed the questionnaire on a computer console, after giving informed consent through the same on-line procedure and reading online instructions. Each questionnaire lasted approximately 30 min.

### Results

#### Inclusion criteria

Since the main aim of the ratings was to ensure the interpretability of the pragmatic sentences in untimed conditions for the purpose of the timed sensicality judgment task, we excluded pragmatic sentences for which both the following criteria were satisfied: (i) median score equal to 1 for the meaningfulness scale and median score > 3 for the difficulty scale; (ii) depending on the set, for metaphors: median score equal to 5 or to 1 for the familiarity scale; for metonymies: cases in which less than 80% of participants correctly associated the producer with the corresponding product; for approximation: approximations for which the adjective-nouns pair scored < 2 (Mdn) on the typicality judgment scale. Literal and anomalous counterparts of the excluded pragmatic sentences were dropped as well.

From the original pool of sentences, 6 triplets were eliminated from each set. Final stimuli comprised 42 triplets for each of the 3 sets, resulting in a total of 378 sentences (see **Supplementary Material**). In the following, we only report rating results for the final pool of sentences, to be further employed in the timed sensicality task.

#### Linguistic measures

Since the target word X was constant in the pragmatic, literal, and anomalous sentences of each triple, length, and frequency were exactly balanced within each set. Length of the target words was also balanced across sets [mean number of characters: metaphor = 7.07; metonymy = 7.16; approximation = 7.49; *F*_(2, 123)_ = 1.42, *p* = 0.24]. Frequency of the target words were controlled for metaphor and approximation [mean log frequency: metaphor = 1.51; approximation = 1.54; *F*_(1, 82)_ = 0.43, *p* = 0.83] based on a 3 million words database of written Italian, fully lemmatized and annotated (Corpus e Lessico di Frequenza dell'Italiano Scritto, CoLFIS, Bertinetto et al., 2005), available through the web interface EsploraCoLFIS (Bambini and Trevisan, 2012). No values were available in the database for the proper names used in the metonymy sets. Ratings collected in the world knowledge task should suffice as a measure of subjective frequency (see below). Overall, average frequency based on the values of all content words of the sentence was balanced across sets [mean log frequency: metaphor = 1.92; metonymy = 1.85; approximation = 2.04; *F*_(2, 123)_ = 1.47, *p* = 0.23].

#### Rating results

We applied nonparametric methods since the assumptions underlying the use of parametric tests were violated in our sets (Knapp, 1990; Jamieson, 2004; Carifio and Perla, 2008; Norman, 2010). For descriptive statistics, we used median as a measure of central tendency and interquartile range as a measure of dispersion. For each set, Kruskal–Wallis chi-squared test was performed on raw data to assess whether there were overall differences across pragmatic, literal and anomalous sentences (meaning modulation factor). As *post-hoc* tests, we used Mann–Whitney U tests with Bonferroni correction for multiple comparisons (true alpha level = 0.0167) to determine which of the three types of sentences differed from each other. We also conducted parametric statistics on rank transformed data (Conover and Iman, 1981). In all cases, the results confirmed those obtained with the nonparametric procedure, and will not be reported in the results section. Table 2 presents descriptive statistics of the key psycholinguistic variables computed for each set.

**Table 2:** **Descriptive statistics of rating scores for the Metaphor set, the Metonymy set, and the Approximation set**.

		**Pragmatic**	**Literal**	**Anomalous**
Metaphor set	Meaningfulness	4 (2–4)	4 (4–5)	1 (1–2)
	Difficulty	2 (1–2)	1 (1–2)	1 (1–2)
	Familiarity	3 (1–4)	4 (3–5)	1 (1–1)
	Cloze probability	0.00%	0.39%	0.00%
Metonymy set	Meaningfulness	4 (3–5)	5 (4–5)	1 (1–2)
	Difficulty	1 (1–2)	1 (1–2)	1 (1–2)
	World knowledge	90.29%	–	–
	Cloze probability	0.00%	0.00%	0.00%
Approximation set	Meaningfulness	4 (3–5)	5 (4–5)	2 (1–2)
	Difficulty	1 (1–2)	1 (1–1)	2 (1–2)
	Typicality	4 (3–4)	5 (4–5)	1 (1–1)
	Cloze probability	1.66%	11.44%	0.39%

*Median and interquartile range (in brackets) are reported for meaningfulness, difficulty, and typicality tasks. Cloze probability and world knowledge results are reported in percentage*.

Furthermore, we complemented the inferential analyses set by set by analyzing meaningfulness and difficulty scores across the three sets through correspondence analysis (PASW Statistics 18.0.0 and R 2.13.0, “languageR” package, Baayen, 2011; see also Baayen, 2008). The correspondence analysis is an explorative computational method for interpreting categorical variables (Greenacre, 1993), and it is capable of uncovering relationships both among and between variables. Thus, it can be especially suitable for testing the relations between categorical variables such as meaningfulness and difficulty.

The correspondence analysis tests the association between two variables organized into a contingency table and seeks to provide a low dimensional map of the association between rows and columns of the contingency table. In the present analysis, all sentence stimuli were in rows, while Likert scores from 1 to 5 were in columns. By means of this analysis, the dimension of the original space is reduced, and an optimal subspace—closest to the cloud of points in the chi square-metric—is found. The information's loss due to the dimension reduction is represented by the inertia explained by the axes of the map. The number of dimensions (hereafter called “factors,” according to Baayen, 2011) needed to explain the variation in the data were determined by using the scree plot. In the scree plot the factors' eigenvalues were plotted in order of magnitude from largest to smallest, and the point where there was a marked drop in the amount of variation explained was considered: only those factors with inertia contribution higher than this point were selected for the interpretation. Hence, the coordinates of both row and column points of the contingency table were projected onto the selected low-dimensional subspace. In this spatial map, row and column points that are close together are more alike than points that are far apart. For each factor, we also plotted the mean coordinates of the points of each sentence type by means of bar plots in order to describe the distribution of points with respect to the different types of sentences across sets. Mean coordinates were then statistically compared.

#### Metaphor set

Both metaphorical and literal sentences scored median 4 on the meaningfulness scale (metaphorical: Mdn = 4, iqr = 2–4; literal: Mdn = 4, iqr = 4–5), while anomalous sentences were rated as meaningless (Mdn = 1, iqr = 1–2). The effect of meaning modulation (three levels: pragmatic, literal, anomalous) was found significant [χ^2^_(2)_ = 432.84, *p* < 0.001]. Metaphorical and literal sentences significantly differed from anomalous counterparts (metaphorical vs. anomalous, *p* < 0.001; literal vs. anomalous, *p* < 0.001). Although both metaphorical and literal scores were in the upper end of the scale, statistically literal sentences resulted more meaningful than metaphorical sentences (literal vs. metaphorical, *p* < 0.001), probably due to a greater variability for metaphors. In all cases, participants formulated their judgments about the sense/nonsense of the sentences with no difficulty (metaphorical: Mdn = 2, iqr = 1–2; literal: Mdn = 1, iqr = 1–2; anomalous: Mdn = 1, iqr = 1–2), although there was an effect of meaning modulation [χ^2^_(2)_ = 29.59, *p* < 0.001], due to higher scores for metaphor (metaphorical vs. literal, *p* < 0.001; metaphorical vs. anomalous, *p* < 0.001). Familiarity ratings showed that metaphorical sentences received medium values (Mdn = 3, iqr = 1–4). Literal sentences scored higher (Mdn = 4, iqr = 3–5), and the difference was significant [χ^2^_(2)_ = 495.71, *p* < 0.001; literal vs. metaphorical, *p* < 0.001]. Cloze probability was very low throughout the set, scoring 0.00% for metaphorical and anomalous sentences, and 0.39% for literal sentences.

#### Metonymy set

Metonymic and literal sentences received high scores on the meaningfulness scale (metonymic: Mdn = 4, iqr = 3–5; literal: Mdn = 5, iqr = 4–5), while anomalous sentences scored median 1 (iqr = 1–2). Meaning modulation yielded a significant effect [χ^2^_(2)_ = 446.02, *p* < 0.001], with metonymic and literal items more meaningful than anomalies (metonymic vs. anomalous, *p* < 0.001; literal vs. anomalous, *p* < 0.001). As in the metaphor set, scores for both the metonymic and the literal items were at the upper end of the scale, but the comparison was significant (literal vs. metonymy, *p* < 0.001). Difficulty was very low across conditions (in all cases, Mdn = 1, iqr = 1–2). Nevertheless, we observed an effect of meaning modulation [χ^2^_(2)_ = 14.69, *p* < 0.001], and the comparison between metonymies and literal sentences was significant (*p* < 0.001). World knowledge task showed that participants correctly associated the producer with the product (accuracy = 90.29%). Cloze probability was 0.00% for any version of any item.

#### Approximation set

On the meaningfulness scale, both approximations and literal sentences received high scores, while anomalous sentences received low scores (approximate: Mdn = 4, iqr = 3–5; literal: Mdn = 5, iqr = 4–5; anomalous: Mdn = 2, iqr = 1–2). A significant effect of meaning modulation was found [χ^2^_(2)_ = 451.60, *p* < 0.001]: both approximation and literal sentences were judged more meaningful than anomalies (approximate vs. anomalous, *p* < 0.001; literal vs. anomalous, *p* < 0.001). Consistently with findings on metaphor and metonymy, literal sentences were more meaningful than approximation sentences (*p* < 0.001). Difficulty was low throughout the set (approximate: Mdn = 1, iqr = 1–2; literal: Mdn = 1, iqr = 1–1; anomalous: Mdn = 2, iqr = 1–2), although the comparison between approximation and literal sentences reached significance (χ^2^_(2)_ = 101.89, *p* < 0.001). Results of the typicality task showed that the adjectives were judged moderately appropriate when referred to the nouns used in the approximations (Mdn = 4, iqr = 3–4), and fully appropriate when referred to the nouns used in the literal sentences (Mdn = 5, iqr = 4–5), while they were rated inappropriate in combination with the nouns from the anomalous sentences (Mdn = 1, iqr = 1–1). All comparisons were significant [χ^2^_(2)_ = 560.61, *p* < 0.001; *p*'s < 0.001]. Cloze probability remained below the threshold of 12%, with averaged values of 1.66% for approximations, 11.44% for literal, and 0.39% for anomalous expressions.

#### Meaningfulness and difficulty across sets

**Figure 1:**
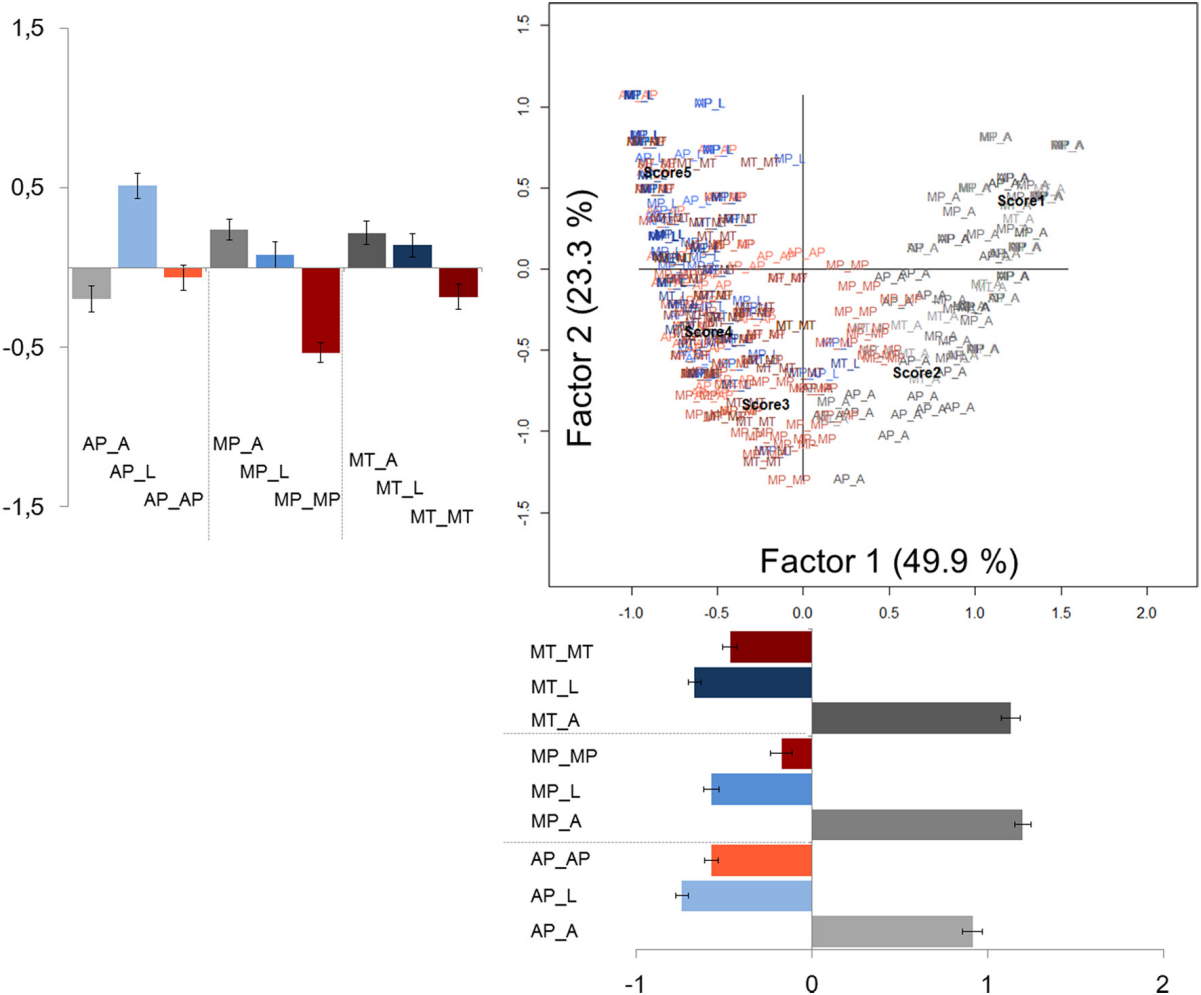
**Correspondence analysis for meaningfulness rating scores**. The 378 sentences belonging to the three sets (Metaphor set = MP; Metonymy set = MT; Approximation set = AP) and the 5 Likert points are plotted at their corresponding coordinates. MP_MP: Metaphor set—metaphorical sentences; MP_L: Metaphor set—literal sentences; MP_A: Metaphor set—anomalous sentences; MT_MT: Metonymy set—metonymic sentences; MT_L: Metonymy set—literal sentences; MT_A: Metonymy set—anomalous sentences; AP_AP: Approximation set—approximate sentences; AP_L: Approximation set—literal sentences; AP_A: Approximation set—anomalous sentences. Pragmatic sentences are shown in magenta (MP_MP = magenta; MT_MT = dark magenta; AP_AP = light magenta); literal sentences are shown in blue (MP_L = blue; MT_L = dark blue; AP_L = light blue); anomalous sentences are shown in gray (MP_A = gray; MT_A = dark gray; AP_L = light gray). Barplots indicate mean coordinates for each factor and sentence types; error bars indicate standard error means.

A synthetic view of the similarity among the different types of sentences across sets with respect to meaningfulness and difficulty is provided through the correspondence analysis. For meaningfulness, a correspondence analysis with all the sentence stimuli across the three sets as one variable and Likert scores as the other variable was performed. A significant model was generated and the chi-squared test revealed a significant association between variables [χ^2^_(1508)_ = 3437.10, *p* < 0.001]. The scree plot revealed a marked decrease in the proportion of inertia explained by the second and subsequent eigenvalues; accordingly, only the first factor was considered to be interpreted. The first factor, accounting for 49.87% of the total inertia, roughly revealed a segregation of the different types of sentences into two clusters: literal and pragmatic sentences belonging to the three sets are on the left side of the map, whereas all anomalous sentences are on the right side (Figure 1). Hence, the first factor seems to indicate that all pragmatic and literal sentences were similarly scored on the meaningfulness scale. Anomalous sentences differed from pragmatic and literal ones, being collocated apart. It can also be observed that, among meaningful sentences, on the one hand literal sentences were clustered together, on the other hand approximate, metonymic and metaphorical sentences were close together. By statistically comparing the mean coordinates along the first factor, a significant difference was found between anomalous and both pragmatic and literal sentences [*F*_(2, 375)_ = 987.40, *p* < 0.001; Bonferroni *post-hoc* comparisons, *ps* < 0.001]. Significant difference was also found between literal and pragmatic sentences (Bonferroni *post-hoc* comparisons, *p* = 0.002). Considering the first factor with respect to the Likert scores, we observed that it was organized from right to left according to the ascending order of the Likert scale values.

**Figure 2:**
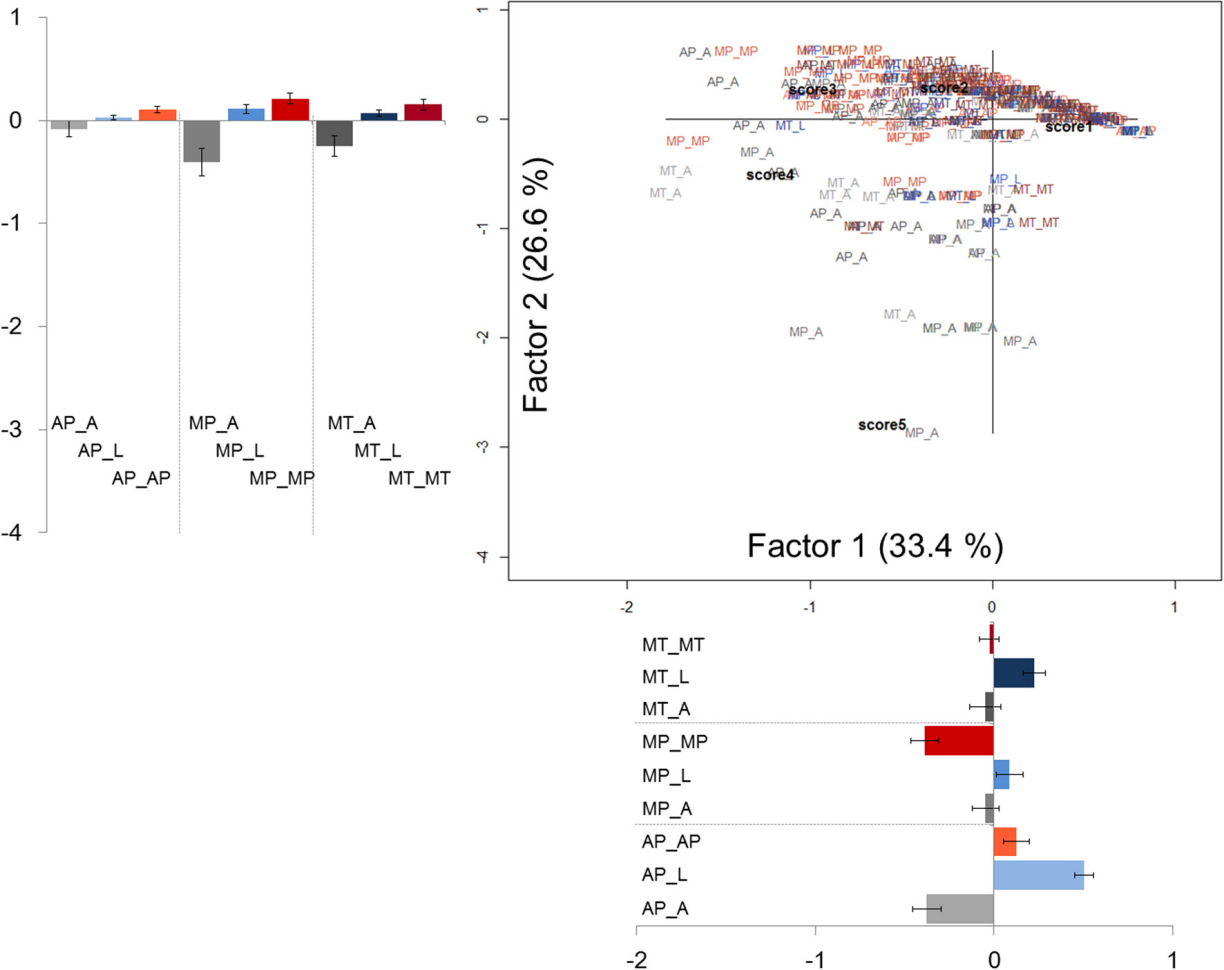
**Correspondence analysis for difficulty rating scores**. Labels as in Figure 1.

The correspondence analysis was also applied to difficulty ratings. A significant model was generated [χ^2^_(1508)_ = 2067.82, *p* < 0.001]. The resulting scree plot revealed a marked decrease in the proportion of inertia explained by the third and subsequent eigenvalues; hence, the first factor (explaining the 33.41% of the total inertia) and the second factor (explaining the 26.61% of the total inertia) were interpreted. As shown in Figure 2, the majority of sentences were close together, clustered in the upper part of the plot, suggesting that sentences were almost perceived as similarly difficult to be understood. However, upon a closer inspection, the first factor seems to reveal a distinction between literal sentences (on the right side) and anomalous ones (on the left side). Pragmatic sentences showed a more dispersed distribution, with metaphors mainly distributed on the left side and approximations mainly distributed on the right side of the map. By statistically comparing the coordinates along the first factor, literal sentences significantly differ from both pragmatic and anomalous sentences [*F*_(2, 375)_ = 27.50, *p* < 0.001; Bonferroni *post-hoc* comparisons: pragmatic vs. literal, *p* < 0.001; anomalous vs. literal, *p* < 0.001]. With respect to the Likert scores, we observed a separation between score 1 and all other score values.

By visually inspecting the second factor, we observed a rough separation of literal and pragmatic sentences (in the upper half of the map) from anomalous sentences (on the bottom half of the map), thus, most likely reflecting the similarity between literal and pragmatic sentences as compared to anomalous ones. This interpretation was further supported by the statistical analysis on the mean coordinates on the second factor [*F*_(2, 375)_ = 27.16, *p* < 0.001; Bonferroni *post-hoc* comparisons: pragmatic vs. anomalous, *p* < 0.001; literal vs. anomalous, *p* < 0.001; pragmatic vs. literal, *p* = 0.38].

### Discussion

Through a rating procedure, we built a set of tightly controlled metaphorical, metonymic, and approximate uses (and literal and anomalous counterparts) based on the same sentence structure “That X verb Y,” characterized in terms of meaningfulness and difficulty. For each set, inferential analysis revealed that both pragmatic and literal sentences were rated as meaningful and differently from anomalous sentences, thus, excluding the possibility that pragmatic items are interpreted as anomalous. Inferential analysis also pointed out differences within the group of meaningful sentences. Although pragmatic sentences were judged interpretable (with high median scores for meaningfulness and low median score for difficulty), literal uses score higher in meaningfulness and lower in difficulty than pragmatic uses. This is probably related to the fact that the context prompting pragmatic mechanisms to emerge is a minimal one. However, these differences do not seem to jeopardize the general consistency of the sets. Accordingly, the correspondence analysis on meaningfulness shows that literal and pragmatic sentences of the three sets clustered together and were judged similarly for meaningfulness, clearly differing from anomalous sentences. It also showed the similarity among all pragmatic uses, as opposed to all literal counterparts. Furthermore, all sentences were judged similarly on the difficulty scale, suggesting that our stimuli were all easily interpretable although some specific characteristics are revealed. All literal sentences, regardless the specific set, resulted overall easy to be interpreted. More importantly, literal and pragmatic uses were similar for difficulty and different from anomalous sentences.

All pragmatic uses were also controlled for familiarity. Metaphorical sentences received medium scores on the familiarity scale, which suggests that they were perceived neither as fully conventionalized nor as extremely creative. Likewise, for metonymy, participants correctly associated the proper names of the producers to the corresponding product, thus, implicitly demonstrating the familiarity of the names and of the metonymic transfer, although not fully lexicalized. For approximation, data suggested that the selected adjectives where judged appropriate when referred to the nouns used in the approximations, yet less typical than when used literally, providing first quantitative evidence for the definition of the category of approximation.

The set of stimuli also appears to be well controlled for the contextual expectancy: cloze probability was very low across sets, never above 12.00% for any condition of any sets. Interestingly, participants never created metaphor nor approximation in completing the sentences. Strictly speaking, also for metonymy the cloze probability was equal to zero. However, we observed 7 cases in which the final word reported by the participants was a proper name, albeit different from the one used in the corresponding stimulus (e.g., original stimulus: “That writer translates *Fruttero*”; cloze probability results: “That writer translates… *Hesse/Sartre*”). These results highlighted that there were some verbs spontaneously used in their metonymic sense, while the probability of creating a metaphor or an approximation was not verified, suggesting that metonymy is somehow more prone to routinization.

Overall, the result of the ratings allows us to assume that potential differences in processing pragmatic and literal sentences in the timed sensicality judgments will not depend on significant psycholinguistic differences of materials, but will truly reflect distinct interpretations for the three phenomena.

As a final note, one may argue that target words had different syntactic functions across sets, being used predicatively in metaphor and approximation, and referentially in the case of metonymy. However, we believe that it was important to focus on standard uses of the three pragmatic phenomena, rather than maintaining the same target word at the price of less clear and prototypical pragmatic types.

## Timed sensicality judgment study

Timed sensicality judgment task has been used as a valuable paradigm to explore interpretation assignment, at different levels of the linguistic structure. This paradigm has been widely employed in investigations targeting conceptual operations, including polysemy (Klein and Murphy, 2001) and compounding (Gagné, 2001). At the sentence level, sensicality judgments have been used to explore pragmatic interpretation of conjunctions (Bott et al., 2009) and processing literal, metaphorical and idiomatic expressions containing actions verbs (Cacciari and Pesciarelli, 2013). The advantage of the sensicality judgment task is that it requires not only to access but also to elaborate the meaning of the expression. Information can be gathered both on the availability of the correct interpretation under time pressure (measured in terms of accuracy, i.e., proportion of correct responses—judging a sensible expression to be sensical or a non-sense expression to be non-sensical) and on the costs of interpretation (measured in terms of latencies). Interestingly, sensicality judgments often recur as behavioral task in a number of experimental paradigms targeting figurative language processing, from Speed-Accuracy Tradeoff (McElree and Nordlie, 1999) to neurophysiological and neuroimaging studies (Arzouan et al., 2007; Lai et al., 2009; Rapp et al., 2011; Subramaniam et al., 2012).

Here we used timed sensicality judgments to explore interpretation assignment of different types of pragmatic uses (i.e., metaphor, metonymy and approximation) compared to literal counterparts. It could be hypothesized that: (i) the greater meaning modulation required by loose uses compared to literal uses might reflect in higher interpretation costs for both metaphorical and approximate expressions compared to literal expressions; (ii) in turn, the greater meaning modulation required by metaphor compared with approximation (broadening + narrowing vs. only narrowing) might reflect in higher interpretation costs for metaphorical relative to approximate expressions; (iii) as for metonymy, being based on a different conceptual operation with respect to metaphor and approximation, and being more subject to routinization, it is possible that no additional interpretation costs are required with respect to literal interpretation.

### Methods

#### Participants

Twenty-five native speakers of Italian (12 M/13 F; mean age = 25.32 ± 3.02 years; mean schooling years = 18.3 ± 3.03) participated in the study. Participants were unaware of the aim of the study, and not experts in linguistics or psycholinguistics. None of them had participated in the rating study. They gave written consent to participate after receiving an explanation of the procedures, according to the Declaration of Helsinki, and received a monetary reimbursement for their participation.

#### Materials

The final pool of sentences described in the previous section was used as stimuli, i.e., 42 triplets for each set (Metaphor, Metonymy, Approximation). An additional 42 anomalous sentences were included for each set, with the purpose of having a similar ratio of sense and non-sense items. In order to minimize potential effects related to the repetition of the target words X in the triplets, the additional items recombined other words in the set, partly by repeating the subject nouns Y (e.g., “Those insects are tables,” where “insects” is the subject noun in the literal version of one triplet in the metaphor set; see Table 1) and partly by repeating the last word of the additional item (e.g., “Those trousers are tables,” where “tables” is the last word of the additional item obtained as above). Furthermore, to reduce the proportion of pragmatically used words and avoid metalinguistic awareness on figurative language, the experimental items were intermixed with 594 fillers (66% sense, 33% non-sense), consisting of four word sentences, like the experimental stimuli. In total, there was a sense:non-sense ratio of 1.44:1, and pragmatic sentences represented 12.0% of the stimuli (4.0% metaphors, 4.0% metonymies, and 4.0% approximations).

#### Procedure

Each participant was tested individually. Stimulus presentation and response collection were all carried out on a personal computer, using Presentation^©^ software (Version 14.9, www.neurobs.com). Each trial began with a fixation cross presented in the middle of the screen for 500 ms. Next, the sentence was presented word by word at a fixed rate (300 ms). After the final word, YES/NO appeared on the screen to indicate that participants could give their response. Participants were instructed to respond as quickly and accurately as possible, and to make a sensicality judgment by pressing the green button when the string was meaningful and the red button when the sentence was meaningless on an RB 530 response pad (SuperLab Pro, Cedrus Corporation). The assignment of red and green to the left and right keys was counterbalanced across participants. After response or time-out (4000 ms), there was a blank inter-trial interval of 1000 ms. Response times were measured from the offset of the target word X.

Each subject was presented with all sentences. To avoid fatigue, three experimental blocks were created. An equal number of pragmatic, literal, and anomalous sentences from each set were included in each block, along with an equal number of fillers. We assigned the members of each triplet and the additional anomalous counterpart to distinct blocks, in order to avoid long-distance priming effects. Within each block, sentences were presented in a random order, while the order of the block was pseudo-randomized across participants. Mandatory stops between experimental blocks were fixed. A training session including 10 items preceded the experiment. Furthermore, two practice trials (not included in the analysis) were administered at the beginning of each block. Overall the experimental session lasted 1 h.

### Results

Responses faster than 250 ms and slower than 1750 ms were excluded from the analysis (10.4% of the data). We also excluded data by two participants with overall accuracy rate lower than 80%, and by one participant with 40% of responses faster than 250 ms. We observed that for the Metaphor set one participant never answered correctly for metaphors. However, he was not excluded from the analysis as his overall level of accuracy was higher than the 80% threshold. Similarly, one metaphorical item was never judged accurately, but it was not excluded from the analysis based on the results of the rating study. In Table 3 accuracy rates and mean reaction times for correct responses for each experimental condition are reported.

**Table 3:** **Accuracy rates and mean reaction times (ms) for correct responses as a function of pragmatic modulation (pragmatic, literal, anomalous conditions) and set type (Metaphor set, Metonymy set, Approximation set)**.

	**Metaphor set**		**Metonymy set**		**Approximation set**	
	**Accuracy**	**Reaction time**	**Accuracy**	**Reaction time**	**Accuracy**	**Reaction time**
Pragmatic	0.52 (0.24)	744.66 (339.02)	0.83 (0.10)	688.70 (338.17)	0.87 (0.09)	655.96 (308.82)
Literal	0.95 (0.04)	638.10 (305.05)	0.89 (0.07)	658.32 (326.55)	0.95 (0.04)	591.13 (293.18)
Anomalous	0.97 (0.05)	653.98 (339.44)	0.95 (0.06)	669.01 (332.04)	0.85 (0.16)	673.37 (333.20)

*Standard deviations in parentheses*.

#### Accuracy

A Univariate General Linear Model with meaning modulation (three levels: pragmatic, literal, anomalous) and set type (three levels: metaphor set, metonymy set, approximation set) as fixed factors was carried out on accuracy rates, treating either subjects (*F*_1_) or items (*F*_2_) as a random factor. Results showed that both the meaning modulation factor [*F*_1 (2, 168)_ = 60.74, *p* < 0.001; *F*_2 (2, 328)_ = 68.98, *p* < 0.001] and the set type factor [*F*_1 (2, 168)_ = 10.74, *p* < 0.001; *F*_2 (2, 328)_ = 12.41, *p* < 0.001] were significant. Also their interaction was significant [*F*_1 (4, 168)_ = 32.33 *p* < 0.001; *F*_2 (4, 328)_ = 37.47, *p* < 0.001], indicating that the effect of one factor depends on the level of the other factor. We therefore, explored the effect of meaning modulation set by set by carrying out a separate ANOVA for each set, with a focus on the comparison between the pragmatic and the literal sentences. In the Metaphor set, this factor yielded significant effects [*F*_1 (2, 42)_ = 68.43, *p* < 0.001; *F*_2 (2, 82)_ = 100.77, *p* < 0.001], with metaphorical sentences being less accurate than literal sentences (Bonferroni *post-hoc* comparisons, *ps* < 0.001 both by subjects and by items). Also in the Metonymy set, meaning modulation was significant [*F*_1 (2, 42)_ = 12.73, *p* < 0.001; *F*_2 (2, 82)_ = 8.10, *p* = 0.001]. In contrast to the Metaphor set, however, accuracy doesn't seem to vary for metonymic and literal sentences: *post-hoc* comparisons revealed only a marginal difference between the two conditions (Bonferroni *post-hoc* comparisons, *p* = 0.05 in the by subject analysis, *p* = 0.18 in the by item analysis). In the Approximation set, again we observed a main effect of meaning modulation [*F*_1 (2, 42)_ = 3.94, *p* = 0.02; *F*_2 (2, 82)_ = 8.49, *p* = 0.001]. Accuracy for approximation was significantly lower than for literal sentences in the by item analysis (Bonferroni *post-hoc* comparisons, *p* = 0.006), although the difference was not significant in the by subject analysis (Bonferroni *post-hoc* comparisons, *p* = 0.12). Overall, the data suggest a higher availability of literal uses as compared to metaphor and—to a lesser degree—approximation, but not for metonymy.

#### Latencies

Following the standard in analyzing response times, only trials in which participants responded correctly were included in the analysis.

**Figure 3:**
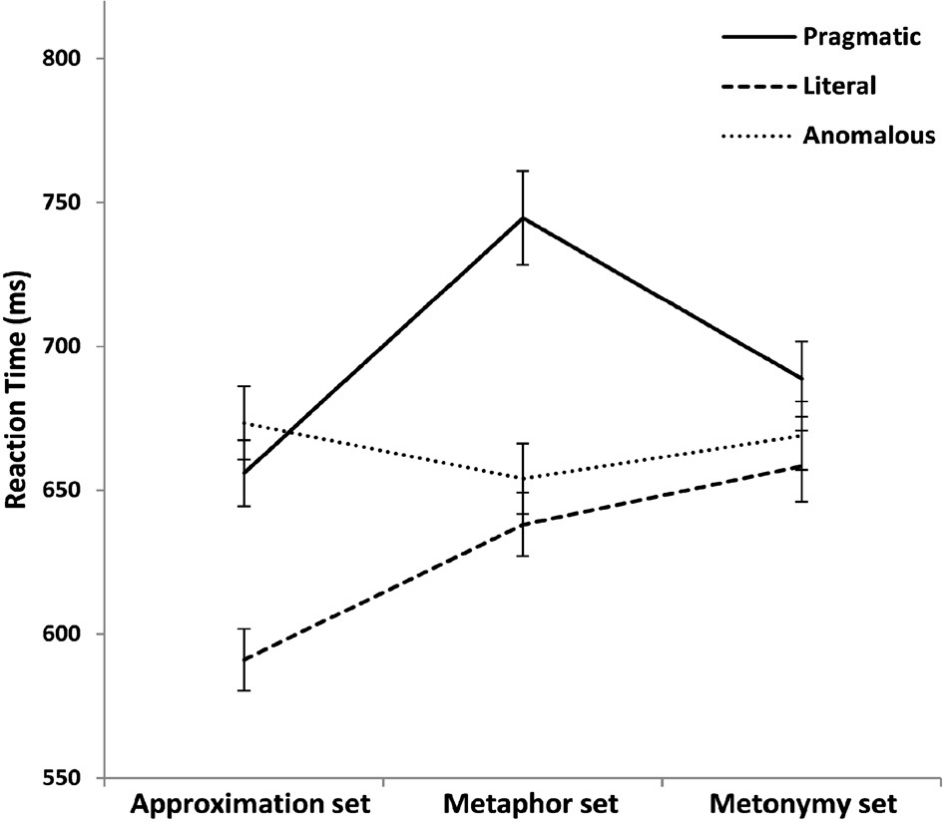
**Mean reaction times (ms) for the Approximation set, the Metaphor set, and the Metonymy set as a function of the meaning modulation factor**. Pragmatic level is represented by the solid line, literal level by the dashed line, and anomalous level by the dotted line. Error bars indicate standard error.

The effect of meaning modulation (three levels: pragmatic vs. literal vs. anomalous) and set type (three levels: Metaphor set, Metonymy set, Approximation set) on response times were examined with Univariate General Linear Model treating either subjects (*F*_1_) or items (*F*_2_) as a random factor. We observed a significant effect of meaning modulation [*F*_1(2, 167)_ = 12.23, *p* < 0.001; *F*_2(2, 327)_ = 24.97, *p* < 0.001], as well as a significant effect of set type in the by item analysis and marginally significant in the by subject analysis [*F*_1(2, 167)_ = 2.95, *p* = 0.05; *F*_2(2, 327)_ = 9.46, *p* < 0.001]. A significant interaction between meaning modulation and set type was found [*F*_1(4, 167)_ = 3.77, *p* = 0.006; *F*_2(4, 327)_ = 8.70, *p* < 0.001], as shown in Figure 3. In order to explore the interaction of meaning modulation and set type, simple effect analyses were conducted. As concerns the meaning modulation factor, in the Metaphor set we observed that metaphorical sentences were interpreted slower than literal counterparts [*F*_1 (2, 41)_ = 5.85, *p* = 0.006; *F*_2 (2, 81)_ = 25.51, *p* < 0.001; Bonferroni *post-hoc* comparisons, *p* < 0.05 in the by subjects, *p* < 0.001 in the by items analysis]. On the contrary, in the Metonymy set there were no differences across conditions [*F*_1 (2, 42)_ = 0.74, *p* = 0.48; *F*_2 (2, 82)_ = 2.06, *p* = 0.13], indicating that metonymic interpretation was reached as rapidly as literal interpretation. Similarly to the Metaphor set, the Approximation set showed a significant effect of the meaning modulation factor [*F*_1 (2, 42)_ = 10.66, *p* < 0.001; *F*_2 (2, 82)_ = 8.37, *p* < 0.001], with approximations interpreted slower than literal sentences (Bonferroni *post-hoc* comparisons, *p* = 0.006 in the by subjects, *p* = 0.003 in the by items analysis).

**Figure 4:**
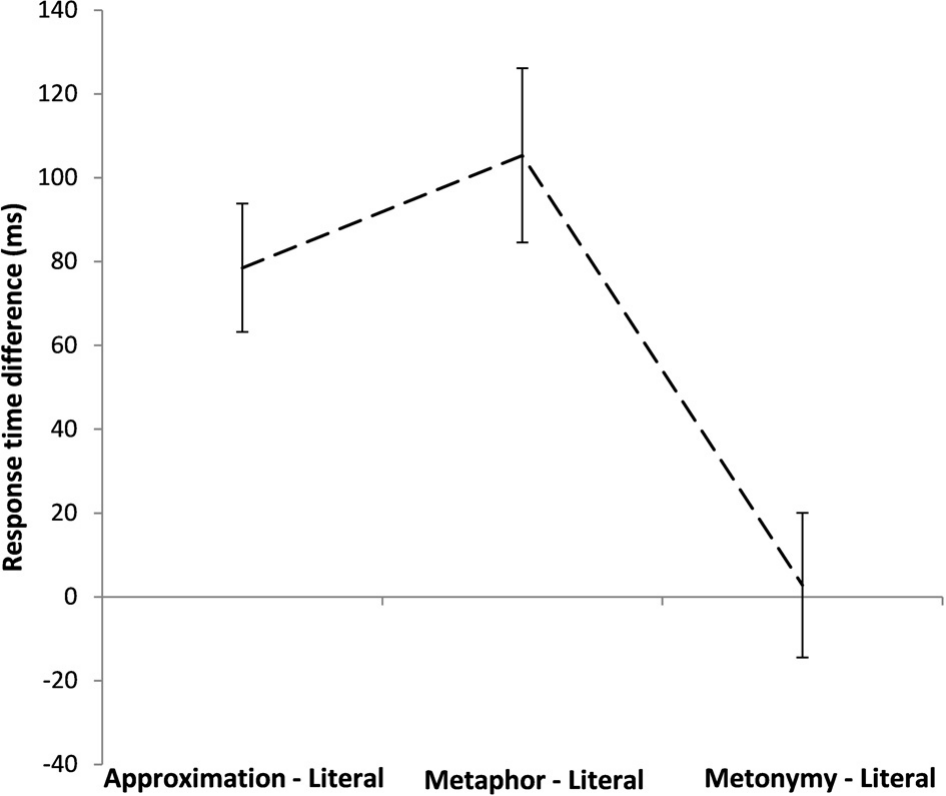
**Reaction times differences (pragmatic minus literal) for the Approximation set, the Metaphor set, and the Metonymy set**. Error bars correspond to the standard error of the difference for each set.

We also assessed whether the type of pragmatic use has an effect on response times. Since pragmatic sentences differ in some respects—as needed to preserve clear pragmatic types, we avoided direct comparisons of metaphors, metonymies, and approximations across sets. Rather, we measured the latency difference between the pragmatic condition and the literal condition for the corrected pairs of each set: (metaphor—literal), (metonymy—literal), and (approximation—literal), as represented in Figure 4. The highest latency difference was obtained for metaphor (*M*_metaphor_−literal = 105.34 ms), followed by approximation (*M*_approximation_−literal = 78.50 ms), while a minimal latency difference was observed for metonymy (*M*_metonymy_−literal = 2.76 ms). The comparison reveals an effect of the type of pragmatic use [*F*_(2, 41)_ = 14.14, *p* < 0.001], with metaphor and approximation significantly different from metonymy, but not different from each other (Bonferroni *post-hoc* comparisons: metaphor/approximation vs. metonymy, *p* = 0.001; metaphor vs. approximation, *p* = 0.92).

## General discussion

The results of the rating study indicated that the three pragmatic uses were easily interpreted as meaningful in a manner similar to the corresponding literal constructions. More specifically, meaningfulness and difficulty ratings pointed toward similar behavior of all pragmatic uses (metaphors, metonymies, approximations) and the literal sentences as opposed to anomalous sentences. Despite some differences in the inferential statistics between literal uses and single pragmatic uses, the correspondence analysis showed that the literal and pragmatic uses behave alike in differing from anomalies. Importantly, the observed difference between the literal and figurative in the inferential analysis was consistent across metaphor, metonymy, and approximation sets, i.e., in all cases pragmatic sentences exhibited a similar behavior as opposed to literal sentences. By characterizing sentences with respect to several psycholinguistic dimensions, we provided measures that may guide the selection of stimuli in future studies (see **Supplementary Material**).

While the rating study indicates similarity in the meaningfulness and difficulty of the various pragmatic uses, timed sensicality judgments revealed that there are differences across metaphor, metonymy, and approximation, reflected both in accuracy rates and response latencies during interpretation. Extending the current theoretical models into experimental predictions, we expected (i) metaphors and approximations to exhibit higher interpretation costs with respect to literal interpretation; (ii) metaphors to be more costly with respect to approximations; and (iii) metonymies to behave differently, possibly in the direction of no additional costs with respect to literal interpretation. Our results seem to confirm these predictions and to show differences across the three pragmatic phenomena, reflected both in accuracy and response times.

First, meaning modulation (i.e., whether the expression was pragmatic, literal, or anomalous) significantly affects the accuracy of the response. Assigning a pragmatic interpretation under the pressure of time seems to be more difficult than constructing a literal interpretation. There are, however, notable differences across pragmatic uses. For metaphor, the percentage of correct responses was around 50%. Although judged as meaningful in the offline rating, in timed sensicality judgments, metaphors proved significantly more difficult to interpret than literal expressions. This piece of evidence is consistent with previous research employing sensicality judgments in a variety of paradigms, from Speed Accuracy Tradeoff (McElree and Nordlie, 1999) to neurophysiological recording (Arzouan et al., 2007) and neuroimaging (Subramaniam et al., 2012). Accuracy around chance seems thus, a common performance associated with the interpretation of nominal metaphor in speeded condition. Participants performed much better when they were presented with approximations and metonymies, reaching 87 and 83%, respectively. This suggests higher availability for approximate and metonymic uses with respect to metaphorical use. Interestingly, we observed that metaphors and approximations—although less clearly—are interpreted less accurately than their literal counterparts, while there is a marginal or no difference between metonymies and literal expressions. This points in the direction of similarities in the interpretation style of metaphor and approximation, although associated with different degrees of availability. In turn, the availability of metonymy seems to equate that of literal interpretation. It should be noted that in the Metonymy set literal sentences reached only 89% accuracy, which is lower than accuracy literal sentences in other sets. This is probably related to the costs of processing proper names, which require the retrieval of stored knowledge (see Gorno-Tempini et al., 1998), also when they are well-known by the participants (as assessed in the rating study). However, this should not affect the comparison between metonymies and literal controls, as proper names were included in both conditions and their presence should not block interpretative differences to emerge, if any. At the same time, there is experimental evidence that common and proper nouns are supported by different functional and anatomical paths (Semenza, 2006). In this sense, the impact of proper nouns on processing metonymical expressions as compared to metaphorical and approximate uses cannot be accounted for in the present study, and may require further investigations.

Second, pragmatic interpretation also reflects in time. Analyzing error-free trials, i.e., limiting the analysis to those cases where pragmatic sentences were judged to be sensical, we observed that interpreting pragmatic uses is not always slower than literal interpretation. This is the case for metaphor and approximation, but not for metonymy. These findings accord with behavioral literature on metaphor showing that, in minimal context and for not conventionalized expressions, metaphor processing requires extra costs compared to literal processing (Noveck et al., 2001). Here, we carefully controlled the sentential environment, by providing each pragmatic use with a minimal context, and the familiarity of the expressions, by avoiding lexicalized cases. Thus, the higher reaction times for metaphors seem to truly reflect extra costs required by the interpretation of metaphorically used words as compared to literal uses.

Results are also consistent with eye-tracking studies reporting no differences between metonymic and literal expressions (Frisson and Pickering, 1999; Pickering et al., 2004). It cannot go unnoticed, however, that, at the neural level, metonymic expressions elicit robust differences from literal comprehension (Rapp et al., 2011; Schumacher, 2011, 2013). It is up to future studies to elucidate whether this discrepancy is motivated by differences in the materials, either in conventionality or supportive context, or in the methodological techniques and the type of information they offer. Our view is that, when metonymy—like in our case—is based on common shifts such as *producer for product*, no matter the relative conventionality of the specific lexical items, in a minimal yet sufficient context, interpretation costs in speeded conditions closely mirror literal comprehension, and radically differ from those required by metaphor processing.

Besides, this view is consistent with findings reported for other types of routinized meaning shift. For example, sensicality judgments on logical metonymy showed no differences in accuracy nor in latencies between the coerced and control conditions, yet again evoking neural differences (Brennan and Pylkkänen, 2008). By contrast, our results seem to be conflicting with the higher reading times for resolving metonymic referring expressions as compared to metaphorical referents presented in Gibbs (1990). However, those data are controversial, as potentially affected by the plausibility of the items (Frisson and Pickering, 1999), and obscuring some comparisons of interest (Noveck et al., 2001).The different patterns observed for metaphor and metonymy gains support from the results on approximation. We showed that adjectives used approximately are interpreted slower than the same adjectives used literally. This piece of evidence seems to place approximation closer to metaphor than to metonymy. This result seems to strengthen the distinction between the processing styles for metaphor and for metonymy, by introducing a third case that patterns like the former but differently from the latter. Consonant with this are also the latency differences between the pragmatic and the literal conditions across sets. When we disentangle the costs of interpreting each type of pragmatic use, we see that approximation and metaphor are associated with extra costs, while metonymy doesn't prompt extra effort. Accuracy data, with metaphor and approximation as well (although to a lesser degree) departing from literality, and metonymy tending to equate it, are in harmony.

Collectively, this pattern of results carries importance for discussing theoretical accounts of the nature of pragmatic phenomena. The relevance-theoretic claim that metaphor and approximation both require conceptual adjusting of the linguistically encoded concept but in different degrees (Wilson, 2003) seems to be supported by our data, and specifically by the gradient observed in availability and latency. Also the direction of the conceptual adjustment may contribute to the different gradient observed in the sensicality judgments. According to Carston and Wearing (2011), hyperbole, is considered as a case of marginal broadening as opposed to metaphor involving a broadening coupled with narrowing. Extending this proposal further, also approximation could be taken as a case of marginal broadening (possibly more marginal than in hyperbole) as opposed to the combination of broadening and narrowing supporting metaphor. According to this interpretation, higher difficulty and costs for metaphor compared to approximation might stem from a more complex operation—broadening and narrowing—with respect to the marginal broadening required for approximation processing. For the type of task used here, we do not have direct evidence to discriminate whether the difference between approximation and metaphor lays in marginal vs. radical broadening or in marginal broadening vs. a combination of radical broadening and narrowing. Intuitively, our data fit well with the degree claim posited by Relevance Theory, while the direction claim is less straightforwardly answerable. More sophisticated designs will be needed that manipulate the (degree of) direction of the adjustment, possibly exploring the temporal dynamics of the process or the conceptual properties that undergo manipulation. Granted this caveat, the general idea of a modulation in the underlying conceptual adjustment process seems to be well supported by our findings. Converging evidence comes from Deamer et al.'s (2010) reading time study, where hyperbolic uses were compared to metaphorical uses, showing that even a more substantial type of broadening such as hyperbole is distinct from metaphor. This study actually failed in finding a difference between hyperboles and literal expressions. This discrepancy is possibly related to contextual modulation: Deamer et al. (2010) used supportive contexts that might have facilitated hyperbole resolution and reduced the broadening, while we used a minimal sentential environment that allowed for the marginal extra costs required by approximation to emerge.

Also the hypothesis put forward in Carston (2010), i.e., that metonymy is not straightforwardly reducible to narrowing or broadening but involves some kind of shift, seems to fit with our data. The different pattern of results observed for metonymy as opposed to metaphor might reflect different conceptual operations. Some support for this interpretation comes from acquisition data showing that metonymy not only is acquired at a faster rate than metaphor, but it also processed more accurately throughout childhood to adulthood (Rundblad and Annaz, 2010). In order to explain this finding, Rundblad and Annaz (2010) hypothesized a more basic type of conceptual operation for metonymy as opposed to metaphor. Similarly, a contiguity vs. property relation was assumed to motivate different behaviors in aphasic patients (Bisiacchi et al., 1976; Semenza et al., 1980). Neuropsychologial data suggested that the processing of class vs. thematic relationships rely, at least in part, on partially independent neural substrates, being associated with posterior and anterior lesions, respectively (Semenza et al., 1992).

The results described for metonymy can also be reconciled with the Cognitive Linguistics account, to the extent that metaphor and metonymy are ascribed to distinct types of mappings: across domains for metaphor, within the same domain for metonymy. A greater cognitive distance between concepts can be assumed for metaphor (see also Rundblad and Annaz, 2010) and might be reflected in higher difficulty and costs. As it is still difficult to translate the different types of mappings in terms of processing costs, we leave this for further research to develop.

Our data also point to reduced efforts for metonymy, and to the routinization of some types of metonymic shifts, such as *producer for product*. It might be of some interest here to report some qualitative insights from the post-experiment session: despite the very low percentage of metaphors in the sentence pool, some participants noticed their presence, while none seemed to notice metonymy, as if metonymic uses were more integrated in the lexical knowledge and less prominent in the speakers' metalinguistic awareness. Interestingly, in a developmental study, Annaz et al. (2009) observed a correlation of metonymic comprehension with the expansion of receptive vocabulary that might suggest that in some cases metonymic meanings might be part of the lexicon.

According to these observations, a possible distinction might be sketched between the combination of broadening and narrowing on the metaphor side, less pre-configured in direction and degree of the conceptual adjustment of the lexical concept, and conceptual shift on the metonymy side, based on more routinized patterns. In other words, metonymy seems to have a more direct relation with the lexical meaning and is much less arbitrary than metaphor. Highly creative metonymic uses are possible as well (consider, for instance, “The best pencils of the world gather together for the annual drawing convention”), and this might call upon higher interpretation costs. However, it seems psychologically implausible to posit different elaboration procedures for the same class of phenomena, as the difference between routinized *producer for product* cases and less typical *tool for worker* cases could probably be made not by different types of conceptual adjustment processes, but rather by the role of context.

As a final consideration, sensicality judgments are a good measure of the availability and difficulty of correct interpretation, but are limited to stages where the sense has already been construed, and do not account for online processing nor for the type of process involved (Frisson, 2009). Thus, our results shed light on the costs of interpretation assignment, and provide insights into interpretative style, but further investigations are needed to explore the temporal dynamics and the nature of the conceptual operations involved.

## Conclusions

Behind a label such as figurative language, many different mechanisms are grouped. Although we assume that all require pragmatic inferencing to be interpreted, interpretation might come with different procedures, linked to different operations at the conceptual level. Through timed sensicality judgments recorded for different pragmatic uses in minimal context condition, we found that there are significant differences in the interpretation availability and costs of metaphor, metonymy, and approximation. The findings support a theoretical distinction between metaphor and approximation, which seem to vary in degree and possibly in the direction of the underlying adjustment process, compatible with Relevance Theory, and an even more marked separation with metonymy, whose meaning shift might be subject to routinization.

With these data, we hope to have strengthened the empirical basis available on figurative language, by providing the first evidence in favor of the psychological reality of the phenomenon of approximation, and with a first attempt to answer the challenge raised by metonymy. We believe that deepening the understanding of the phenomena included under the realm of pragmatics, by pinpointing potential differences for the parser and elaborating on whether natural classes of cases can be identified on this basis, is one promising line of research for the experimental pragmatics enterprise.

## Conflict of interest statement

The authors declare that the research was conducted in the absence of any commercial or financial relationships that could be construed as a potential conflict of interest.
